# Modify the Histone to Win the Battle: Chromatin Dynamics in Plant–Pathogen Interactions

**DOI:** 10.3389/fpls.2018.00355

**Published:** 2018-03-19

**Authors:** Juan S. Ramirez-Prado, Sophie J. M. Piquerez, Abdelhafid Bendahmane, Heribert Hirt, Cécile Raynaud, Moussa Benhamed

**Affiliations:** ^1^Biological and Environmental Science and Engineering Division, King Abdullah University of Science and Technology, Thuwal, Saudi Arabia; ^2^Centre National de la Recherche Scientifique, Institut National de la Recherche Agronomique, University Paris-Sud, University of Évry Val d’Essonne, University Paris Diderot, Sorbonne Paris-Cite, University of Paris-Saclay, UMR9213 Institut des Sciences des Plantes de Paris Saclay, Essonne, France

**Keywords:** plant immunity, plant pathogen, chromatin, histone modifications, post-translational modification, virulence

## Abstract

Relying on an immune system comes with a high energetic cost for plants. Defense responses in these organisms are therefore highly regulated and fine-tuned, permitting them to respond pertinently to the attack of a microbial pathogen. In recent years, the importance of the physical modification of chromatin, a highly organized structure composed of genomic DNA and its interacting proteins, has become evident in the research field of plant–pathogen interactions. Several processes, including DNA methylation, changes in histone density and variants, and various histone modifications, have been described as regulators of various developmental and defense responses. Herein, we review the state of the art in the epigenomic aspects of plant immunity, focusing on chromatin modifications, chromatin modifiers, and their physiological consequences. In addition, we explore the exciting field of understanding how plant pathogens have adapted to manipulate the plant epigenomic regulation in order to weaken their immune system and thrive in their host, as well as how histone modifications in eukaryotic pathogens are involved in the regulation of their virulence.

## Introduction

As sessile organisms, plants must face a plethora of unavoidable stressors, ranging from extreme temperatures to nutritional deficiencies, chemical toxicity, herbivores, and pathogen attacks. As a consequence, they have acquired several sophisticated regulatory mechanisms that allow them to cope with such adverse conditions. The epigenetic regulation of the plant genome, or the physical modification of chromatin, is a highly dynamic process that fine-tunes the expression of a pertinent set of genes under certain environmental or developmental conditions.

In plants, as in other eukaryotes, stress responses involve sequentially stress detection, signaling, differential gene expression and a physiological outcome, regulated in a temporal fashion (modified from Gambino and Pantaleo, 2017). Stress-sensing mechanisms have been deeply studied and characterized in plants, as well as their physiological outcome; however, there are still many challenges toward the understanding of stress-activated signaling pathways and how are they linked to specific transcriptional changes in the nucleus.

In the eukaryotic nucleus, DNA is wrapped around histone oligomers, forming highly complex structures by interacting with several other proteins; this DNA–protein complex is defined as chromatin. Chromatin can sustain a wealth of post-translational modifications (PTMs) that physically regulate the accessibility of the transcriptional machinery to certain genomic regions, making loci more or less permissive for transcription. Such PTMs do not only impinge on DNA accessibility, but also on the recruitment of specific proteins involved in several processes, including transcription, DNA replication and repair. Histones cannot only be modified, but also replaced by histone variants with different physical properties, or released, in order to allow gene expression, DNA repair or replication (Kouzarides, 2007; Eichten et al., 2014).

As it can be expected, the chromatin PTMs profile is very dynamic; a characteristic that can be attributed to the activity of several histone-modifying proteins and complexes that mediate the addition, deletion, reading and maintenance of these marks. Plenty of chromatin modifications have been identified in eukaryotic cells, including DNA methylation, as well as histone acetylation, methylation, phosphorylation, ubiquitination, sumoylation, carbonylation, and glycosylation, among others (Kouzarides, 2007; Tan et al., 2011). Some histone marks are generally associated with specific transcriptional states: for instance, acetylation and methylation in lysine 4 of histone 3 (H3K4me3 and H3K4me1) are linked to transcriptionally active genes (Marmorstein and Roth, 2001; Carrozza et al., 2003; Shlyueva et al., 2014), whereas the tri-methylation of the lysine 27 (H3K27me3) is commonly associated to the transcriptional silencing of repressed genes (Lindroth et al., 2004; Schubert et al., 2006). Other marks, including H3K9me2 and H3K9me3, are mainly enriched in heterochromatic regions such as chromocenters and regions with high density of transposable elements (TEs), where they have a constitutive repressive function (Stewart et al., 2005).

In the recent years, numerous studies have been performed toward the characterization of the epigenomic regulation of stress responses in plants. Several publications have described how diverse biotic and abiotic stresses affect chromatin PTMs, with their respective transcriptional and physiological implications. Furthermore, there is a significant amount of published reviews addressing the topic; however, most of them focus on DNA methylation and/or abiotic stresses (Luo et al., 2012; Kim et al., 2015; Pandey et al., 2016; Secco et al., 2017). For this reason, in this review, we aim to summarize and discuss the current knowledge regarding the role of histone modifications in the regulation of plant–pathogen interactions, a relatively unexplored area of research with exciting findings and promising perspectives.

## The Defense: Plant Responses to Biotic Stress From a Chromatin Perspective

The processes and molecules that participate in biotic stress responses in plants have been exhaustively studied. Thus, it is generally accepted that these organisms can respond to pathogens either in a PAMP- (pathogen-associated molecular pattern) or an effector-dependent manner, as it was first described in the zigzag model proposed by Jones and Dangl (2006). On the other hand, higher plants, including *Arabidopsis thaliana*, rice and wheat, contain various pathogen-responsive MAPKs, which transduce cytoplasmic signals upon pathogen detection (Meng and Zhang, 2013; Xu et al., 2014) and lead to the activation of transcription factors and a hormone signaling cascades that regulate the expression of well-characterized defense-related genes (Pieterse et al., 2009; Birkenbihl et al., 2012). Various plant hormones participate in such immune responses, which in general are divided into two different pathways: one mediated mainly by salicylic acid (SA) and the other one by jasmonic acid (JA) and ethylene (ET). Generally, the SA-mediated pathway is effective against biotrophic pathogens, microorganisms that get their energy from the living cells of their host and keep them alive. On the other hand, the JA/ET pathway works against pathogens with a necrotrophic lifestyle: microorganisms that feed from the debris of the dead cells of their hosts, which they kill through diverse means (Glazebrook, 2005; Sorokan et al., 2013).

It has become clearer in the last years that plant immunity is a much more complex system than the generally accepted models. A large-scale study, performed by Lewis et al. (2015), depicted the dynamics of the Arabidopsis transcriptional response to *Pseudomonas syringae* pv. *tomato* (*Pst*) DC3000, a bacterial pathogen causing the speck of tomato that also infects crucifers. Through the infiltration of adult plants with the *Pst* DC3000 strain and its non-pathogenic mutant *hrpA* (defective in effector delivery to the plant cell), the authors were able to determine that most of the defense- or disease-related genes, are induced within 6 h post-infection, prior to pathogen replication (Lewis et al., 2015). Interestingly, the two bacterial strains modulate the expression of different sets of genes, attributed to the capacity of *Pst* DC3000 to deliver its repertoire of about 28 effectors into the host cell, inducing effector-triggered susceptibility (ETS) through the manipulation of the host cell activity, including gene expression (Lewis et al., 2015). Likewise, the interaction between the SA- and JA/ET-mediated immunity pathways is highly affected by levels and signaling of other hormonal compounds and molecules, including auxin, cytokinins, brassinosteroids, and gibberellins. Such interactions lead in some cases to non-completely antagonistic scenarios between the JA/ET and SA pathways, allowing the plant to fine-tune its response in a pathogen-specific manner (Halim et al., 2006; Fu and Wang, 2011; De Bruyne et al., 2014; Naseem et al., 2015; Xia et al., 2015).

Several studies have depicted that chromatin regulation plays a major role in the expression of defense-related genes, and the establishment of a fast and appropriate physiological immune response (Ding and Wang, 2015). Indeed, several Arabidopsis and rice lines with mutations in genes involved in histone acetylation, methylation, ubiquitination and chromatin remodeling, show altered resistance levels to diverse microbial pathogens. These results, further discussed below, indicate the importance of the epigenomic machinery in several plant physiological processes, including immune responses.

### Histone Acetylation in Defense Responses

All 4 core histones can be acetylated and deacetylated in different positions, producing 26 putative acetylation sites on a single nucleosome (Lusser et al., 2001). Lysine residues located primarily on N-terminal histone tails can be acetylated on the following positions: 9, 14, 18, and 23 of histone 3 and 5, 8, 12, 16, and 20 of histone 4. Recently, it has been reported that H2A and can be also acetylated (Hartl et al., 2017). In general, Acetylation affects chromatin by negatively perturbing the interactions between nucleosomes, leading to a looser chromatin state, and permitting the binding of proteins involved in transcription (Berger, 2007; Shahbazian and Grunstein, 2007).

The antagonistic activity of histone acetyltransferases (HATs) and histone deacetylases (HDACs) is responsible for histone acetylation levels. HATs attach the acetyl moiety of acetyl-CoA to the lysine amino group, while HDACs remove acetyl group from histones (Hassig and Schreibert, 1997; Ma et al., 2013). Each eukaryotic genome encodes several of these enzymes, and their expression dynamics and specificity differ significantly. For instance, the expression of some rice HDACs is induced by abiotic stresses, such as cold and drought, while others are repressed by the same stimuli (Hu et al., 2009).

The Arabidopsis HDA19 has been one of the most studied and characterized plant HDACs. Its role in biotic stress responses is evidenced by the increased susceptibility of the *hda19* knock-down mutant to the necrotrophic pathogen *Alternaria brassicicola*. The enzyme was assigned to the JA-dependent pathway since its expression increased upon exposure to JA, ET and to the studied fungus. Consequently, the mutant displays decreased expression of genes that participate in the JA defense (Zhou et al., 2005), while overexpressor lines present an increased resistance to necrotrophic pathogens, indicating that HDA19 is a positive regulator of defense against these microorganisms (Zhou et al., 2010). This HDAC has also been reported to be important for the repression of SA-mediated defense responses: loss of HDA19 leads to increased SA levels and increased expression of SA-defense markers (including *PR1* and *PR2*), even in unchallenged plants. In the *hda19* mutant, *PR1 and PR2* display hyperacetylation of H3 (Choi et al., 2012), indicating that HDA19 is important for the removal of acetylation marks and repression of these loci.

In an initial study, the *hda19* mutant was reported to display a reduced resistance to *Pst* DC3000, a pathogen that is controlled mainly via the SA-defense pathway (Kim J.M. et al., 2008). However, it was found in a more recent publication that in fact, the mutant is more resistant to the infection by this bacteria, a coherent phenotype with the high SA levels and expression of SA-defense markers that it displays (Choi et al., 2012). Coherently to the latter result, Kim J.M. et al. (2008) found that HDA19 interacts with two DNA-binding transcription factors from the WRKY family, identified as WRKY38 and WRKY62. Similar to *hda19*, the *wrky38* and *wrky62* mutants presented increased resistance to *Pst* DC3000, evidenced through the development of fewer disease symptoms and a reduced bacterial growth. In addition, WRKY38 and WRKY62 have been shown to be important for the repression of SA-mediated basal defense in Arabidopsis, as it was previously demonstrated for HDA19 (Kim K.C. et al., 2008; Choi et al., 2012).

**FIGURE 1:**
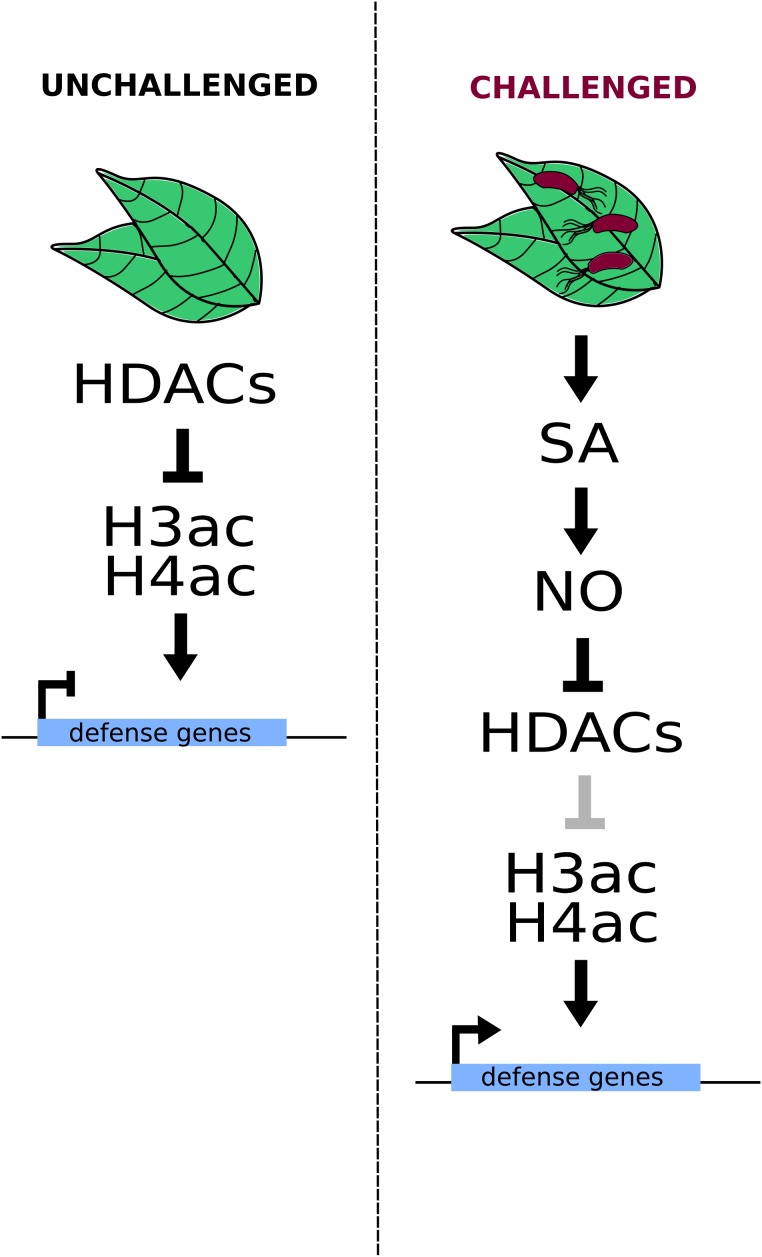
Nitric oxide (NO) induces HDAC inhibition in a SA-dependent fashion. Increased SA (salicylic acid) levels induce NO biosynthesis and the consequent inhibition of histone deacetylases (HDACs), including HDA19. Such inhibition leads to increased H3 and H4 acetylation levels on stress-responsive genes, inducing their transcriptional activation.

Regulation of HDAC activity upon pathogen attacks appears to be mediated at least in part by nitric oxide (NO) (Mengel et al., 2017). This signaling molecule is involved in the biosynthesis, catabolism, transport, signaling perception, and transduction of various hormones (including ET, SA, and JA) (Freschi, 2013), and has been recently reported to affect the expression of biotic and abiotic stress responsive genes in Arabidopsis through the inhibition of HDACs. The treatment of Arabidopsis plants with the NO-donor *S*-nitroso-glutathione (GSNO) led to an increase in the H3 and H4 acetylation levels at several loci that were found to be enriched in cold and defense response genes. Furthermore, the authors demonstrated that SA or INA (a SA functional analog) treatment of Arabidopsis protoplasts induces an increase in NO concentrations, which simultaneously has an effect over histone acetylation through the chemical inhibition of HDACs, including HDA19. In fact, the authors found that upon SA treatment, HDA19 suffers a cysteine oxidation that inhibits its function and that many (≈12.5%) of the NO-regulated H3K9/K14ac sites correspond to HDA19 targets. Therefore, the authors proposed that the activation of the SA-defense pathway leads to the inhibition of HDA19, the consequent hyperacetylation of its target loci, and ultimately, their overexpression (Mengel et al., 2017) (**Figure 1**).

Other HDACs have been described as positive or negative regulators of plant immunity. For example, HDA6 is involved in the JA-pathway, since its mutant and RNAi lines present down-regulation of JA responsive genes, including *PDF1.2, VSP2, JIN1* and *ERF1.* Similar to *HDA19*, *HDA6* expression is induced by JA and ACC (1-aminocyclopropane-1-carboxylic acid, an ethylene precursor) and is important for the repression of defense genes involved in the SA pathway: the mutant displays a constitutive upregulation of SA-defense markers such as *PR1, PR2, EFR, FRK1, WRKY18, WRKY70*, and *WRKY38.* HDA6 has been proposed to function redundantly with HDA19 in the repression of development genes such as the *Flowering Locus C* (*FLC*) and embryo-specific genes; however, it has become more evident that they are also redundant in the regulation of defense, since the *hda6* mutants also display increased resistance to *Pst* DC3000 (Wu et al., 2008).

**FIGURE 2:**
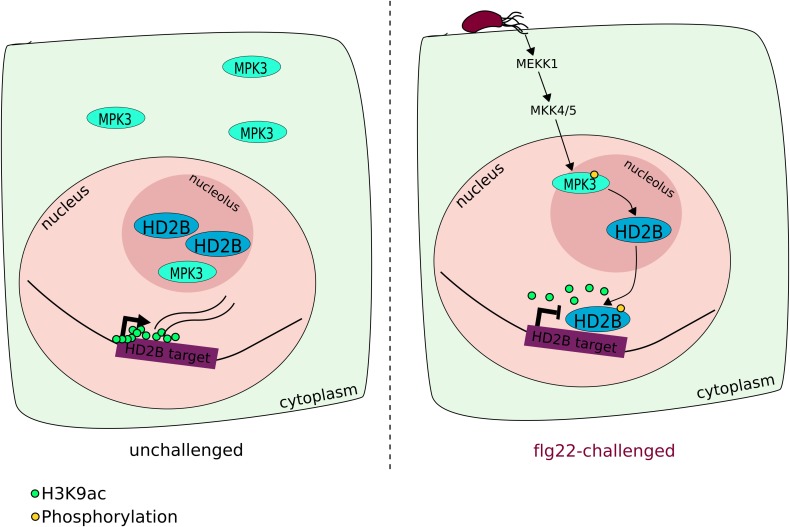
HD2B mediates flg22-induced transcriptional reprogramming downstream of a phosphorylation pathway. flg22 detection activates a well-described MAP kinase phosphorylation cascade that targets MPK3, within other kinases. Once phosphorylated, MPK3 phosphorylates the HDAC HD2B, which relocates from the nucleolus to the nucleus and removes acetylation marks from H3K9 in a genome-wide fashion and contributes to the reprogramming of stress-responsive genes.

By contrast, a MAPK-activated HDAC, HD2B, was shown to positively regulate Arabidopsis innate immunity through reprogramming of defense gene expression upon pathogen detection (Latrasse et al., 2017). MPK3, a well-characterized MAPK involved in the phosphorylation cascade activated by the flagellin peptide, was shown to physically interact with and phosphorylate HD2B. Upon phosphorylation, HD2B mobilizes from the nucleolus to the nucleoplasm, where it removes H3K9ac marks in several loci, thereby fine-tuning the expression of defense genes (**Figure 2**). The authors highlight a few defense-related genes which are HD2B targets and for which promoters get deacetylated upon flg22 treatment, leading to their final reduced expression. The *hd2b* mutant and the mutant line complemented with the non-phosphorylatable version of the protein (HD2B-AA), displayed increased susceptibility to the *hrcC* strain of *Pst* DC3000 (also defective in effector translocation into the plant cell), highlighting the importance of HD2B and its phosphorylation for an appropriate basal immune response. In fact, the authors found that HD2B is a major contributor in the Arabidopsis acetylome changes in response to flg22, since 60% of the hypoacetylated genes upon flg22 exposure are HD2B targets (Latrasse et al., 2017).

Finally, an Arabidopsis homolog of the yeast Sir2 deacetylase, SRT2, was shown to negatively regulate immunity. *srt2* mutants show improved resistance to *Pst* DC3000 and led to increased expression of several loci involved in the SA-defense pathway, including *PR1, PAD4, EDS5* and *SID2*. On the contrary, the overexpressor line is deficient for the induction of *PR1* after pathogen infection, and presents higher bacteria proliferation than the wild type (Wang et al., 2010). Similar studies have been performed in other plants, including crops: for instance, the rice H4 deacetylase HDT701, belonging to the plant-specific HD2 family of HDACs, is overexpressed upon the compatible interaction with the fungal pathogen *Magnaporthe oryzae* (Ding et al., 2012). Overexpression of *HDT701* increases plant susceptibility to both *M. oryzae* and the bacterial pathogen *Xanthomonas oryzae* pv. *oryzae (Xoo)*, and decreases H4 acetylation levels genome-wide. On the other hand, *HDT701* silencing leads to increased H4 acetylation levels and overexpression of pattern recognition receptors (PRRs) and other defense genes. The silenced lines also display increased levels of reactive oxygen species (ROS) after PAMP treatment, and an enhanced resistance to both, *M. oryzae* and *Xoo.* The authors thus conclude that HDT701 is a negative regulator of basal defense in rice (Ding et al., 2012).

Similar to HDACs, HATs also play a role in the regulation of plant defense responses. The Arabidopsis histone acetyltransferase 1 (HAC1) is crucial for the priming of defense genes after environmental stress (Singh P. et al., 2014). Initially, in this interesting publication, the authors described how the challenging of Arabidopsis with diverse abiotic stresses, including heat, cold and high salinity induced chromatin modifications on several PTI-responsive genes (*WRKY53, FRK1*, and *NHL10*) without modifying their expression. After these stresses, the studied loci presented an enrichment of histone marks related to transcriptional activation (H3K9ac, H3K14ac, H3K4me2, and H3K4me3) and a more open chromatin state than in the unchallenged plants. Moreover, the previously stressed plants presented higher resistance to *Pst* DC3000 *hrcC* when infected, accompanied by a higher gene expression of the primed loci and an increased callose deposition. However, the authors also found that even if *hac1-1* mutant displayed wild type levels of resistance, the PTI-responsive genes were not primed in these plants; therefore such mutant did not display any stress-induced increased resistance. In conclusion, even if HAC1 is not directly involved in the expression of defense genes, it is crucial for the priming of PTI genes in response to abiotic stresses (Singh P. et al., 2014).

Another chromatin modifier, the Elongator complex, has been exhaustively studied by Mou and collaborators for its role in plant immunity and histone acetylation. Elongator was initially identified as a protein complex associated with the RNA polymerase II: this complex facilitates transcription by modifying the chromatin in a co-transcriptional manner. Several mutants deficient for Elongator subunits present developmental abnormalities and altered defense responses. Initially, the authors described the Elongator Subunit 2 (AtELP2) as an accelerator of immune responses in Arabidopsis, since the *Atelp2* mutant presents a delayed and reduced defense response (Defraia et al., 2010). Moreover, the authors proved that ELP2 regulates cytosine methylation and histone acetylation levels on several defense genes, including several involved in responses to necrotrophic pathogens, such as *Botrytis cinerea* and *A. brassicicola*. The *Atelp2* mutant presents compromised resistance levels to the studied fungi, together with decreased histone acetylation and expression of JA/ET-defense genes *WRKY33, ORA59* and *PDF1.2* (Wang et al., 2013, 2015). Similar to *Atelp2*, the *Atelp3-10* mutant displays compromised resistance to pathogens: AtELP3 is the catalytic subunit of the Elongator complex and presents two domains necessary for maximum pathogen resistance, a radical *S*-adenosylmethionine domain and a HAT domain, indicating that the acetyltransferase activity of the Elongator plays a role in the regulation of defense responses (Defraia et al., 2013). More recently, several Elongator subunit mutants were reported to present a compromised non-host resistance to the citrus and bean pathogens *Xanthomonas citri* subsp. *citri* (*Xcc*) and *Pseudomonas syringae* pv. *phaseolicola (Psp)* NPS3121 (An et al., 2017). This observation is particularly interesting, since non-host resistance is thought to be a highly durable and multigene trait (Gill et al., 2015). However, in this scenario, the mutation of a single protein is leading to its complete abolishment. Such result implies that the Elongator complex is a major player in the regulation of non-host resistance; nevertheless further research needs to be performed toward the understanding of this relatively unexplored phenomenon. So far no studies have been published regarding genome-wide changes in DNA methylation and histone PTMs in diverse Elongator mutants. Correlating this information with high-throughput transcriptomic data would be a big step toward deepening our understanding in the role of the Elongator in host and non-host resistance in plants.

Histone acetylation thus plays a key role in response to various pathogens. How HAT and HDACs are targeted to the proper loci to allow genome-wide changes in gene expression in response to pathogens remains to be fully elucidated. Interestingly, the EIN2 protein involved in ethylene signaling has recently been shown to modulate histone acetylation in the context of hypocotyl growth: the authors postulate that during ethylene response, the C-terminal domain of EIN2 could be translocated to the nucleus where it would bind to target genes and recruit HATs, resulting in elevating H3K14Ac and H3K23Ac deposition (Zhang et al., 2017). A similar mechanism might be at work, notably during response to necrotrophic pathogens in which ethylene is known to play a role.

### Histone Methylation and the Regulation of Defense

Similar to histone acetylation, histone methylation levels depend on the activity of two types of enzymes with opposite functions. On one side, histone methyltransferases (HMTs) add methyl groups on lysine or arginine residues, while histone demethylases (HDMs) carry out their removal. At variance with histone acetylation, which is considered as a permissive mark for gene expression regardless of the modified residue, histone methylation can activate or inhibit gene expression depending on its position: H3K9me and H3K27me are repressive marks whereas H3K4me and H3K36me are activating marks (Xiao et al., 2016). In addition, histone methylation can occur as mono-, di- and tri-methylations (referred to as me1, me2, and me3), modifications that present different physical properties. As the acetylome, the histone methylome is highly dynamic and regulates a plethora of cellular and physiological processes, involved in development and stress responses. For instance, deposition of H3K4me3 was found to be important for the induction of drought-responsive genes (Alvarez-Venegas et al., 2007; Kim J.M. et al., 2008; Van Dijk et al., 2010). Lysine methylation has been the best-characterized histone methylation mark, and more than 30 histone lysine methyltransferases (HKMTs) have been described in Arabidopsis, which perform methylations on lysines 4 (K4), 9 (K9), 27 (K27), and 36 (K36) of histone 3 (Liu et al., 2010; Pontivianne et al., 2010).

#### H3K9 Methylation

**FIGURE 3:**
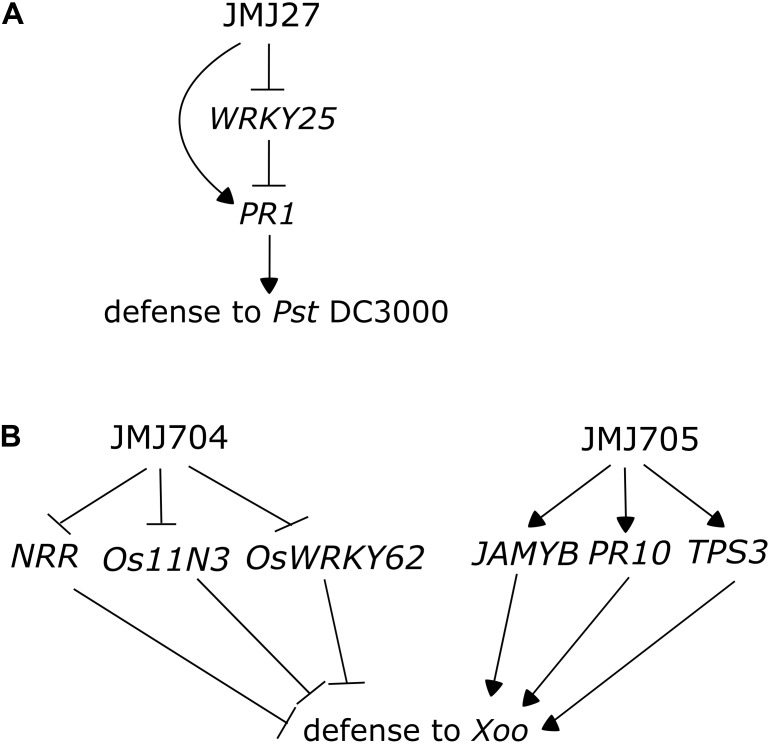
Jumonji C demethylases participate in the regulation of immunity in Arabidopsis and rice. **(A)** Arabidopsis JMJ27 negatively regulates the expression of the *WRKY25* transcription factor, a negative regulator of immunity, and directly or indirectly induces the expression of *PR1*, leading to enhanced resistance to *Pseudomonas syringae* pv. *tomato* DC3000 (adapted from Dutta et al., 2017). **(B)** JMJ704 and JMJ705 contribute to rice resistance to *Xoo* (*Xanthomonas oryzae* pv. *oryzae*) by different mechanisms. JMJ704 represses the expression of negative regulators of immunity *NRR*, *Os11N3*, and *OsWRKY62* by removing activating H3K4me2/3 marks in these loci (adapted from Hou et al., 2015), while JMJ705 promotes the expression of JA-inducible genes *JAMYB*, *PR10*, and *TPS3* through the removal of H3K27me3.

Like acetylation marks, some histone methylations can be associated with specific gene activities and epigenomic states. For instance in plants, as in other eukaryotes, the mono- and dimethylation of lysine 9 of histone 3 (H3K9me1 and H3K9me2) are mainly located in constitutive heterochromatin, where they have a repressive effect (Jackson et al., 2004; Fuchs et al., 2006). Particularly, H3K9me2 has been associated with the repression of repetitive sequences and TEs that can have detrimental effects on the genome when they are reactivated (Lippman et al., 2004). On the other hand, H3K9me3, a much less abundant mark in plants than in animals, locates mainly on the euchromatic loci, rich in protein-coding genes (Mathieu et al., 2005; Turck et al., 2007). The deposition of H3K9 methylation marks has been associated with DNA methylation, since reductions in H3K9me levels are often correlated to a decrease in non-CG methylation; likewise, H3K9 methylation has also been reported to be reinforced by DNA methylation via a positive feedback, since mutants defective in DNA methylation also display reduced levels of histone methylation (Jackson et al., 2002; Johnson et al., 2002; Malagnac et al., 2002; Soppe et al., 2002; Tariq et al., 2003; Mathieu et al., 2005; Vaillant and Paszkowski, 2007; Liu et al., 2010). Jumonji C demethylases, proteins that perform the removal of several methylation marks, including H3K4me2/3, H3K27me3, and H3K9m1/2/3, have been linked with defense responses in rice and Arabidopsis (Hou et al., 2015; Dutta et al., 2017). JMJ27, an Arabidopsis H3K9me1/2 demethylase is a positive regulator of immunity: its mutation increases susceptibility to *Pst* DC3000 and leads to the upregulation of *WRKY25* and *WRKY33*, transcription factors involved in the repression of defense responses (**Figure 3A**) (Dutta et al., 2017).

**FIGURE 4:**
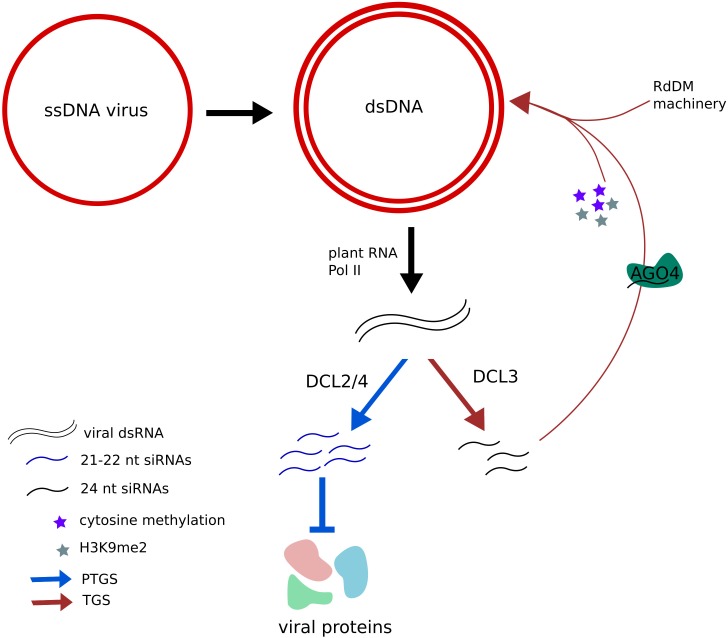
Plants use post-transcriptional gene silencing (PTGS) and transcriptional gene silencing (TGS) as protection mechanisms against virus. Geminivirus uses the plant transcriptional machinery in order to synthesize viral RNAs and produce viral proteins. However, viral transcription can lead to the formation of dsRNAs (double-stranded RNAs) that are diced by DCL2 and DCL4 proteins into 24 nt siRNAs (siRNAs), inhibiting their translation. Such dsRNAs can also be diced into 21–22 nt siRNAs by DCL3 and charged into AGO4 proteins, directing the RdDM (RNA-directed DNA methylation) machinery to the viral genome, where repressive DNA methylation and histone methylation marks are deposited, inhibiting its expression.

Plants protect themselves from many viruses through the RNA silencing pathway, a very sophisticated system based on post-transcriptional gene silencing (PTGS) that targets transcripts from both DNA and RNA viruses (Wieczorek and Obręepalska-Stęeplowska, 2015). The members of the *Geminiviridae* family replicate their single-stranded circular DNA genome using their host replicative machinery. During their replication process, a viral double stranded DNA chain is formed, which is associated with eukaryotic histones, forming small chromosome-like structures. Interestingly, plants also take advantage of transcriptional gene silencing (TGS) in order to defend themselves from viral attacks: they use their DNA and histone methylation machinery in order to turn off viral replication (**Figure 4**). In fact, Arabidopsis mutants deficient in DNA methylation also present reduced H3K9me2 levels in the viral genome upon infection, and hyper-susceptibility to geminiviruses (Raja et al., 2008). A more recent study showed how PolIV and PolV, plant-specific polymerases involved in the canonical RNA-directed DNA methylation (RdDm) pathway, are essential for H3K9me2 methylation but not for DNA-methylation of the geminivirus genome that is performed by a pathway involving RNA PolII and RDR6. This finding indicates that DNA methylation *per se* is not sufficient for the recruitment of H3K9 methyltransferases and the subsequent control of viral replication (Jackel et al., 2016).

#### H3K27 Methylation

While H3K27me1 is located mainly in constitutive heterochromatin in plants, H3K27me3 is important for the repression of many protein-coding genes involved in development and is deposited by the polycomb repressive complex 2 (PRC2). In Arabidopsis MEDEA (MEA), CURLY LEAF (CLF), and SWINGER (SWN), three SET-containing proteins, are thought to have methyltransferase activity and their differential association with different PRC2 elements leads to methylation of different subsets of genes involved in specific developmental processes (Wang et al., 2016). LIKE HETEROCHROMATIN PROTEIN 1 (LHP1), a subunit of the polycomb repressive complex 1 (PRC1) that binds to H3K27me3 and is important for its spreading (Veluchamy et al., 2016), has been found to interact with an heterogeneous nuclear ribonucleoprotein LIF2 (LHP1-interacting factor 2) (Latrasse et al., 2011). Interestingly, LIF2 and LHP1 were found to target many common genes, where they have a general antagonistic role in their activation and repression. However, there is a common set of genes down-regulated in both mutants, enriched in stress response-associated GO terms, that are synergistically regulated by both proteins (Molitor et al., 2016). LIF2 was found to directly participate in the regulation of immunity in Arabidopsis. *lif2* mutant displays an upregulation of SA-mediated defense markers and reduced susceptibility to *Pst* DC3000; however, it is more susceptible to the necrotrophic pathogen *Sclerotinia sclerotiorum*, a phenotype coherent with the down-regulation that it presents in genes involved in JA-response (Le Roux et al., 2014).

JMJ705, a rice demethylase, has been associated with the regulation of defense responses (Li et al., 2013). The overexpressor line of JMJ705 demethylase presents low H3K27me3 levels on various defense genes, including *POX5, POX8, POX22, LOX, AOS2, OPR7, JAMYB, PR5*, and *PR10*, leading to their increased expression, and an enhanced resistance to *Xoo.* Additionally, such lines display a much higher induction in the expression of JA-responsive genes in response to meJA treatment, a phenomenon occurring due to the JA-induced demethylation of the studied loci. In addition to conferring increased resistance to the bacterial pathogen, the overexpression of *JMJ705* leads to a leaf lesion mimic phenotype in mature unchallenged plants: one of the costs of presenting constitutively active defenses (**Figure 3B**) (Li et al., 2013).

#### H3K4 and H3K36 Methylation

The methylation of H3K4 is present among different phyla of the eukaryotic domain and is performed by proteins from the Trithorax group (TrxG). ARABIDOPSIS TRITHORAX 1 (ATX1) is a homolog of the animal H3K4 methyltransferase TRX and has been proven to be involved in various processes, including flowering and defense (Alvarez-Venegas et al., 2003; Alvarez-Venegas and Avramova, 2005). The *atx1* mutant presents a significant genome-wide reduction in H3K4me2 and H3K4me3 levels with several pleiotropic effects, such as flower organ abnormalities, altered flowering time and defense levels (Alvarez-Venegas and Avramova, 2005). *atx1* mutant exhibits a down-regulation of the expression of *PR1* and *WRKY70*, a transcription factor identified as a node of convergence between JA- and SA-defense pathways. This phenotype is accompanied by a slightly increased susceptibility to *Pst* DC3000 *hrcC*, indicating that ATX1 participates in basal defense. ATX1 directly binds the *WRKY70* locus, activating its expression; however, the activation of *PR1* may be occurring indirectly, through WRKY70 activity (Alvarez-Venegas et al., 2007).

Demethylases involved in the removal of H3K4 methylation marks have also been associated to the regulation of defense in plants: JMJ704 was described as a positive regulator of defense in rice and its mutant presents increased susceptibility to *Xoo.* JMJ704 loss of function leads to increased levels in H3K4me2/3 in genes encoding negative regulators of defense, as *NRR, OsWRKY62 and Os-11N3*, leading to their upregulation, and a consequential suppression of defense responses (Hou et al., 2015). Similar to Arabidopsis JMJ27, JMJ704 represses the expression of negative defense regulator; however, its mode of action seems to be different from the one of JMJ705, which activates positive regulators (**Figure 3B**).

Flowering Locus D (FLD or RSI1) was first described as a regulator of flowering time, negatively regulating the well described flowering inhibitor FLC (Liu et al., 2007); however, some recent studies have described it as an important element in the plant response to the signaling that induces systemic acquired resistance (SAR), an enhanced resistance that plants present after a second pathogen challenge, even in previously inoculated organs (Conrath, 2006). Even if the *rsi1* mutant is able to accumulate the SAR-inducing signal, it is incapable of responding to it and accumulating distal SA. This phenomenon leads to a very interesting phenotype, where the mutant does not present a compromised local resistance to *Pst* DC3000 but a compromised SAR (Singh et al., 2013). The biochemical function of FLD has not been characterized; however, it is predicted to play a crucial role in the epigenomics regulation of SAR. Singh et al. (2013) proved that FLD is important for the priming of *WRKY6* and *WRKY29*. Indeed the *rsi1* mutant presents lower levels of H3K4me2 in the promoters of these genes, and fails to show the increase of these marks at these positions, that is observed in WT plants upon SAR induction. These low promoters levels of H3K4me2 have been correlated to a reduced expression of both *WRKY6* and *WRKY29* which have been previously reported to be important regulators of immunity (Singh V. et al., 2014). *WRKY6* was described as a positive regulator of the expression of *PR1* and *NPR1* (Bonardi et al., 2011), and the overexpression of *WRKY29* constitutively activates defense responses in Arabidopsis (Asai et al., 2002).

A 2010 study characterized the role of the Arabidopsis SET DOMAIN GROUP methyltransferase SDG8 (also named EFS, ASHH2, LAZ2, and CCR1; and homolog of the Drosophila ASH1) in immunity: the *sdg8* mutant presents increased susceptibility to *A. brassicicola* and *B. cinerea.* Even though the mutant did not display affected JA levels, it showed impaired expression of JA-responsive genes and misregulation of *MKK3* and *MKK5*, two kinases involved in the phosphorylation cascade activated upon pathogen detection (Dóczi et al., 2007; Rasmussen et al., 2012). Several loci, including *PDF1.2* and *VSP2*, presented lower levels of H3K36me3 in the mutant compared to the wild type. Furthermore, these levels remained unchanged in the mutant upon fungal infection, while they significantly increased in the wild-type plants. Conversely, H3K36me1 levels in *PDF1.2* and *VSP2* decreased in the wild-type following pathogen exposure, while they remained unchanged in the mutant. Similarly, both *MKK5* and *MKK3* loci displayed increased H3K36me3 and decreased H3K36me1 in the wild-type background but not in the *sdg8* mutant in response to infection. These observations provide evidence for the role of SDG8 in the deposition of H3K36me3 and in the induction of its target loci upon pathogen infection (Berr et al., 2010).

More recently, SDG8 was further characterized, together with another methyltransferase, SDG25. Both proteins play a crucial role in diverse mechanisms involved in immunity, since their mutants present impaired PTI, SAR and increased susceptibility to *Pst* DC3000, *B. cinerea* and *A. brassicicola.* In fact, similar to *sdg8* mutant*, sdg25* presents a misregulation in the expression of defense genes and markers in response to the infection of the fungal and bacterial pathogen, including chitinases, glucanases, peroxidases, defensins, *PR1*, *PDF1.2*, and *BIK1* (Botrytis-induced kinase 1). The authors proposed that the role of SDG8 and SDG25 in immunity might be, at least partially, to regulate *CCR2* and *CER3*, genes involved in carotenoid and cuticle biosynthesis, respectively. Both loci were found to present differential down-regulation levels in the *sdg8* and *sdg25* single and double mutants, and the T-DNA insertion mutants of *ccr2* and *cer3* present similar susceptibility levels to *B. cinerea* and *A. brassicicola* to those of *sdg8* and *sdg25* single and double mutants. Furthermore, *sdg8, sdg25, ccr2*, and *cer3* mutants contained lower levels of lipids and cuticular wax, together with increased cuticle permeability, which may be associated to their higher pathogen susceptibility. However, SDG8 and SDG25 seem to have different molecular functions, since SDG8 appears to perform the deposition of H3K36me2 and H3K36me3, while SDG25 may deposit H3K4me1 (Lee et al., 2016).

Further evidence for the role of histone methylation in the immune response comes from the characterization of the Arabidopsis homologs of *ASH1*, *ASHR1*, and *ASHR3*. The authors found that *ashr1* mutation leads to an increased chlorotic lesion area in response to *Pst* DC3000 and the non-pathogenic strain *hrpA*, while the *ashr3* mutant displays reduced symptoms (De-La-Peña et al., 2012). It is important to highlight that in this publication authors described the fast appearing symptomatology in the *ashr1* mutant as hypersensitive response (HR). In this case, the use of the term HR to describe the observed phenomena is not accurate, since this concept refers to an effector-induced cell death that leads to complete resistance. Rather, their work shows that *ashr1* displays enhanced susceptibility to *hrpA*, whereas bacterial growth remains unchanged in the *ashr3* mutant. Authors also observed changes in the deposition of various histone methylation marks in the mutants. For instance, both *ashr1* and *ashr3* mutants present a global deregulation of H3K4me3 and H3K36me2 levels in response to the infection by *Pst* DC3000 and *hrpA.* Interestingly, both mutants also display significantly elevated levels of H3K27me2, which may indicate that they negatively regulate the deposition of this heterochromatic mark during biotic stress responses. In the same publication, the authors observe an upregulation in the expression of *AHR1* and *ASHR3*, as well as *SDG8*, in response to *hrpA*, but a down-regulation of these three genes after the infection with the virulent strain *Pst* DC3000. From this result, they propose that in response to the virulent pathogen the plant down-regulates the expression of these enzymes in order to form what they call “more heterochromatic structures” (De-La-Peña et al., 2012). However, this may instead suggest that the upregulation of these loci is part of the Arabidopsis defense response to the infection, and that the repertoire of T3SS effectors of the virulent bacteria is suppressing their expression in order to promote host susceptibility. This hypothesis appears to be coherent with the increased susceptibility to this bacterium of the *ashr1* and *sdg8* mutants; nonetheless, and in contrast to SDG8, the exact biochemical function of ASHR1 and ASHR3 is still not clear, as well as the definition of its primary targets. Further research need to be performed on the topic in order to clarify their role in plant immunity.

Overall, the available data highlights the critical role of histone methylation both for the repression and the activation of target genes upon pathogen recognition, and the multiplicity of the histone HMT and HDM involved. The challenge for the years to come will be to decipher how specific enzymes are recruited at specific loci, and how their activity contributes to plant short term and long term defense responses.

### Histone Ubiquitination and Defense

Ubiquitination results from the action of three consecutive enzymes adding a single or multiple ubiquitin groups to lysine residues of target proteins (Schnell and Hicke, 2003). Histone proteins can be mono-ubiquitinated on lysine residues and, similar to histone acetylation, histone mono-ubiquitination is usually linked to transcription activation (Weake and Workman, 2008).

H2B mono-ubiquitination is orchestrated in Arabidopsis by two RING E3 ubiquitin ligase enzymes, HUB1 and HUB2 (Cao et al., 2008). HUB1 was identified in Arabidopsis as required for resistance to various necrotrophic fungal pathogens by regulating ET- and SA-mediated responses (Dhawan et al., 2009). In tomato, both HUB1 and HUB2 contribute to disease resistance against *B. cinerea* by modulating the balance between SA- and JA/ET-mediated signaling pathways (Zhang et al., 2015). Little is known about the way HUB1 is specifically addressed to certain loci, such as hormone signaling genes, but HUB1 was proven to interact with MED21, a subunit of the mediator complex, which itself regulates the RNA Polymerase II complex (Dhawan et al., 2009; Zhang et al., 2015). Interestingly Arabidopsis *med21* mutants show the same increased susceptibility to *B. cinerea* as the *hub1* mutants, whereas tomato *med21* mutants do not show any altered disease susceptibility phenotype toward this pathogen (Dhawan et al., 2009; Zhang et al., 2015). It would be highly interesting to test whether, and to what extent, SA-related defense genes are misregulated in the tomato *med21* mutant, which is more resistant to *Pst* DC3000 (Zhang et al., 2015).

**FIGURE 5:**
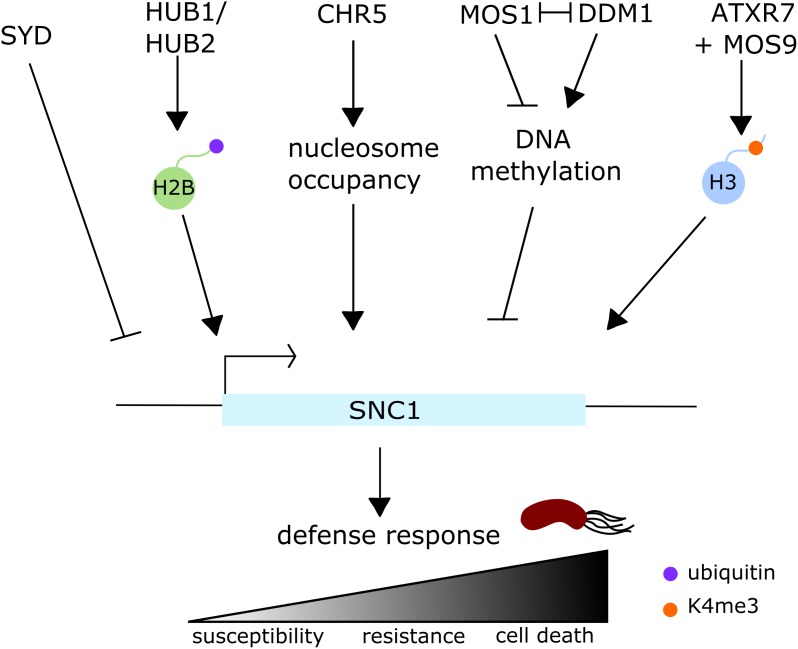
Regulation of the expression of the resistance gene *SNC1* in *Arabidopsis thaliana*. *SNC1* expression is regulated at several levels by multiple histone modifications, nucleosome occupancy and DNA methylation, and its expression levels directly correlate to resistance levels to the bacterial pathogen *Pst* DC3000. On one hand, HUB1 and HUB2, two RING E3 ubiquitin ligases, positively regulate *SNC1* expression via H2B monoubiquitination, while ATXR7, in conjunction with MOS9, positively regulates *SNC1* expression via H3K4 trimethylation. The chromatin remodeler CHR5 also positively regulates the expression of this locus by altering nucleosome occupancy. On the other hand, *SNC1* expression is negatively regulated by at least two chromatin remodelers, SYD and DDM1: it is still unclear how SYD regulates gene expression, but it also appears to negatively regulate the expression of a few SA- and JA/ET-related defense genes. DDM1 negatively regulates *SNC1* expression by methylating its promoter, behaving as an antagonist of MOS1.

Surprisingly, even though Arabidopsis *hub* mutants do not display differential susceptibility to *Pst* DC3000, HUB1 and HUB2 regulate the expression of at least three resistance (R) genes, *SNC1* and two R genes, within the RPP5 cluster (Dhawan et al., 2009; Zou et al., 2014) (**Figure 5**). Fine-tuning of R gene expression is vital for the plant as misregulation of R genes often leads to autoimmune phenotypes, characterized by the inappropriate activation of defenses which results in cell death (van Wersch et al., 2016). *snc1* is a well described autoimmune mutant, caused by a gain-of-function mutation within the TIR-NB-LRR R gene *SNC1* (Li et al., 2001; Zhang et al., 2003). The *bon1* mutation was identified as a suppressor of *snc1* phenotype and BON1 was found to be a negative regulator of SNC1, but similar to *snc1*, the *bon1* mutant itself forms spontaneous lesions (Yang, 2004; Yang et al., 2006). Given that *hub1* and *hub2* mutations partially rescue the autoimmune phenotypes of *snc1* and *bon1* mutants (Zou et al., 2014), it suggests that H2B mono-ubiquitination is responsible for lesion formation. Indeed in such mutants, *SNC1* gene upregulation is linked with an enrichment of H2Bub at this specific locus.

There is a significant crosstalk occurring between histone ubiquitination and other histone modifications – especially histone methylation. For instance, the early flowering *hub1-4* mutant displays reduced levels of H3 methylation (H3K4me3 and H3K36me2) at the flowering genes *FLC*, *MAF1*, *MAF4*, and *MAF5*, suggesting a role of H2Bub for enhancement of H3 hypermethylation (Cao et al., 2008). In a similar fashion, H2B deubiquitination is required for H3K9 dimethylation and heterochromatin formation (Sridhar et al., 2007). Along these lines, but this time in an immunity context, Lee et al. (2016) recently showed that some methylation marks are altered at defense related genes in *hub1-6* mutant background: H3K4me2 levels are reduced at the *SNC1* locus (**Figure 5**). More importantly they showed that H2Bub levels were reduced in mutants deficient in histone methylation. Indeed, the two HMTs mutants *sdg8* and *sdg25*, that are more susceptible to *B. cinerea* infection, also show significantly reduced levels of H3K4me2/3 and H3K36me3 at the defense-related genes *SNC1*, *CER3*, and *CCR2* (Lee et al., 2016). In addition, MOS9, another interesting suppressor of *snc1* was found to interact with the HMT ATXR7 (Xia et al., 2013) (**Figure 5**). *atxr7* mutant can also partially rescue *snc1* lesion mimic phenotype. Interestingly, Xia et al. (2013) showed that *SCN1* expression is also regulated through the trimethylation of H3K4 mediated by ATXR7 and MOS9.

Unexpectedly, H2Bub is also involved in regulating the dynamics of microtubules during the defense response to toxins produced by the necrotrophic pathogen *Verticillium dahliae* (Hu et al., 2014). Despite the lack of epigenomic approaches in this study, *hub1* and *hub2* mutants showed striking phenotypes of delayed microtubules depolymerization following toxins treatment, a phenotype that can be reverted by adding phosphatase inhibitors. In addition to the regulation of phosphatase genes, the authors concluded that microtubules are manipulated by the pathogen by involving H2Bub and protein tyrosine dephosphorylation.

### Chromatin Remodelers in Plant Immunity

Chromatin dynamics is not only orchestrated by histone-modifying enzymes but also by ATP-dependent chromatin remodeling complexes (CRCs). CRCs contain a catalytic sub-unit harboring a conserved SNF2 ATPase domain, but also adjacent additional domains that make each CRC unique and specific (Mohrmann and Verrijzer, 2005). CRCs are helicase-like enzymes that use the energy generated by ATP hydrolysis to alter chromatin structure by sliding, ejecting but also editing nucleosomes by installing and removing histone variants (Clapier et al., 2017). About 40 of such CRCs exist in Arabidopsis, and are categorized into subfamilies based on similarity and ATPase subunits (Flaus et al., 2006; Knizewski et al., 2008). While most of the plant CRCs are examined for their role in development, only five of them have been involved in plant immunity, SYD, DDM1, PIE1, CHR5, and BRHIS1 (Chen et al., 2017).

BRM and SYD are the best described CRCs in plants. They are the respective homologs of the Drosophila BRAHMA and the yeast SNF2, part of the SNF2 subfamily and identified initially for their role in flowering (Wagner and Meyerowitz, 2002; Farrona, 2004). SYD and BRM have undoubtedly overlapping functions based on developmental phenotypes of the respective mutants and because the expression of some genes are equally affected in both mutants (Bezhani et al., 2007). However, they also regulate specific stress signaling pathways. BRM is involved in drought stress response and ABA-signaling but has not been clearly linked to biotic stress. However, the misregulation of *PR* genes in the *brm101* mutant could indicate a role for this protein in SA-related gene regulation (Bezhani et al., 2007; Han et al., 2012; Peirats-Llobet et al., 2016). As for SYD, it specifically regulates the expression of JA/ET responsive genes, by binding – in most cases – to their promoters (Walley et al., 2008). As expected from such gene regulation, the *syd-2* mutant is more susceptible to *B. cinerea* infection (Walley et al., 2008). *syd* mutants do not show altered susceptibility phenotypes to the biotrophic pathogen *Hyaloperonospora arabidopsidis*, but some *syd* alleles show enhanced disease resistance to virulent *P. syringae* (Walley et al., 2008; Johnson et al., 2015). In those lines, SYD was shown to negatively regulate *snc1*-mediated immunity by controlling *SNC1* expression as well as a few other SA- and JA/ET-related defense genes (Johnson et al., 2015) (**Figure 5**). Nonetheless, the mechanism by which SYD regulates the expression of specific defense genes is still to be unraveled. DDM1, another relatively well described CRC because of its conserved SNF2 ATPase domain, is required to maintain DNA methylation even though it has no methyltransferase activity itself (Vongs et al., 1993; Kakutani et al., 1995). DDM1 controls R genes, in particular *SNC1* (Stokes et al., 2002; Yi and Richards, 2007, 2009) (**Figure 5**). DDM1 regulates the expression of the plant resistance gene *SNC1* antagonistically to MOS1, by regulating the methylation levels in the upstream region of the gene (Li et al., 2010). *ddm1* mutation enhances both *snc1* and *bon1* growth defects and *SNC1* expression in these backgrounds, but does not affect their autoimmune phenotypes (Zou et al., 2017). As mentioned above, *SNC1* expression regulation is very complex. It turned out that another chromatin remodeler, CHR5, from the Chd1 subfamily, is a positive regulator of *SNC1* expression (Zou et al., 2017). *chr5* can rescue both *snc1* and *bon1* lesion mimic phenotypes, however, CHR5 acts independently of DDM1 and HUB1 (Zou et al., 2017) (**Figure 5**). CHR5 likely has a more general role in immunity as *chr5* mutant is more susceptible to virulent, avirulent, and non-virulent bacterial pathogens and exhibits impaired nucleosome occupancy at the promoters of some genes (Shen et al., 2015; Zou et al., 2017).

As an increasing number of studies are published on chromatin remodeling, the interplay between chromatin remodelers and histone variants becomes apparent. PIE1 for instance is actively studied for its interaction with the histone variant H2A.Z (Mizuguchi, 2004; Deal et al., 2007; March-Díaz et al., 2008; Berriri et al., 2016). PIE1 is the ATPase of the SWR1 CRC that comprises other non-catalytic subunits such as ARP6, SWC6, SEF (Noh and Amasino, 2003; Deal, 2005; March-Diaz et al., 2006; Martin-Trillo, 2006; Lázaro et al., 2008). The SWR1 complex appears to be important for the defense response; however, the exact role of its different subunits is slightly debatable. March-Díaz et al. (2008) showed that the inactivation of PIE1 and SEF2, as well as H2A.Z, resulted in constitutive activation of defense responses: the mutants display over-expression of many SA biosynthetic and responsive genes and spontaneous cell death in normal conditions, leading to increased resistance to *Pst* DC3000. But it was recently shown that these other SWR1 complex subunits have different functions. Berriri et al. (2016) showed that *pie1*, *swc6* and *hta9 hta11* mutants display severely compromised resistance whereas *arp6* mutant displays increased resistance to the virulent bacterium *Pst* DC3000. Additionally PIE1 and SWR6C play a positive role in ETI induced by various avirulent *Pst* DC3000 effectors but also to the necrotroph *B. cinerea*, while ARP6 does not appear to be involved. As for H2A.Z, mutants lacking this histone variant showed enhanced susceptibility to *B. cinerea* but no ETI phenotype. Altogether these results suggest that the subunits of SWR1 complex play different functions in different defense pathways, and that PIE1 may act in the regulation of the crosstalk between SA- and JA-dependent signaling pathways.

Another level of interplay has been recently identified, between a chromatin remodeler, histone variants and histone monoubiquitination (H2Bub). BRHIS1 is a putative ATPase that belongs to the RAD5/16 subfamily, specific to plants and fungi. In rice, BRHIS1 acts as a negative regulator of defense priming by binding to monoubiquitinated H2A and H2B variants (Li et al., 2015): BRHIS1-RNAi plants are more resistant to infection by *M. oryzae*, while BRHIS1 over-expressors are more susceptible. BRHIS1 represses the expression of defense related genes in normal conditions. These genes are enriched for H2A and H2B variants in their promoters but they are poised for expression activation; after pathogen perception, those poised genes get expressed (Li et al., 2015). At this time, whether the RING finger domain of BRHIS1 does ubiquitinate H2A and H2B remains to be tested.

## The Counterattack: Plant Pathogens also Modify Plant Chromatin (and their Own)

Due to the importance of the epigenomic regulation in the control of plant immunity, and to the long evolutionary competition between plants and their respective pathogens, it has not been surprising to find that plant pathogen can take advantage of their hosts chromatin regulatory network in order to succeed in their infection. In the recent years, plant pathologists, who investigate specific pathosystems into detail, have reported remarkable examples of such mechanisms that are discussed below.

### Plant Transcription Modulation by Plant Pathogens

**FIGURE 6:**
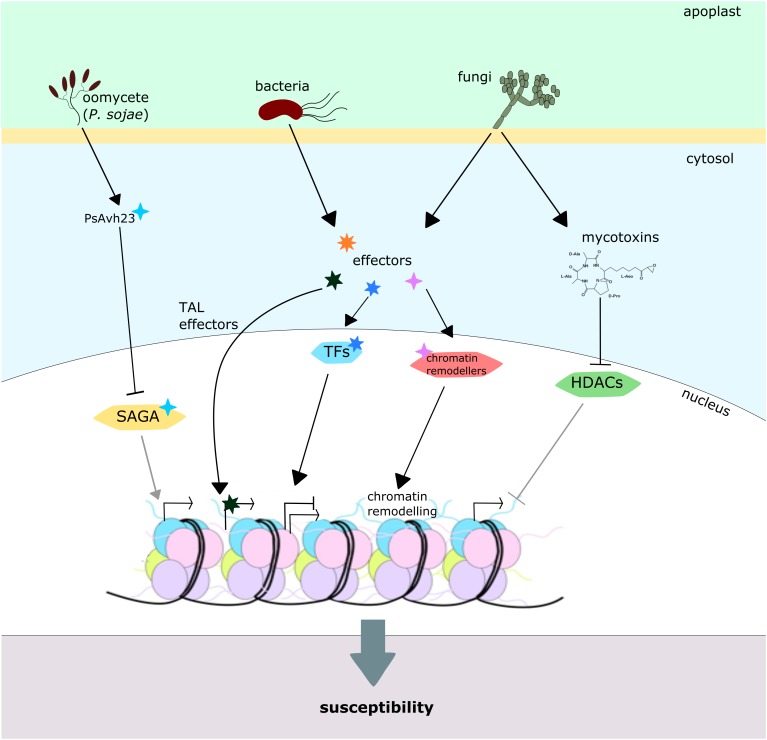
Plant chromatin modification by pathogens. Pathogens from diverse phyla directly or indirectly modify host chromatin in order to promote susceptibility. The *Phytophthora sojae* effector PsAvh23 inhibits the soybean SAGA acetyltransferase complex, reducing genome-wide H3K9 acetylation levels and expression of plenty of defense genes. Likewise, several other bacterial effectors affect plant chromatin by different mechanisms: TAL effectors from *Xanthomonas* spp. and *Ralstonia* spp. locate in the plant cell nucleus and directly activate transcription of target loci, while some other effectors target transcription factors and chromatin remodelers, affecting their activity. Some fungal toxins, depudecin and HC toxin, are capable of inhibiting HDAC activity, promoting histone hyperacetylation and maximal virulence.

It has been known for a while that some mycotoxins, produced by fungal pathogens, can alter the action of certain plant HDACs. This is the case of the HC toxin of *Cochliobolus carbonum*, which inhibits the HDAC activity in maize *in vitro* and *in vivo*, leading to hyperacetylation of H3.1, H3.2, H3.3, and H4, and maximum susceptibility (Brosch et al., 1995; Ransom and Walton, 1997). Similarly, depudecin toxin from *A. brassicicola* was found to inhibit HDACs and to play a role in virulence against cabbage, since depudecin-defective mutants produced smaller lesions than the wild-type strain (Kwon et al., 1998; Privalsky, 1998; Wight et al., 2009) (**Figure 6**). However, downstream molecular consequences of the effect of these toxins *in planta* remain unknown, and the scientific community has focused mainly on the characterization of effector proteins as the main virulence factors.

Transcription activator-like (TAL) effectors from bacteria from the genus *Xanthomonas* spp. and *Ralstonia* spp. have already become classical examples of effectors that target chromatin. These proteins accumulate in the plant nucleus and act as eukaryotic transcriptional activators of specific loci. Through this mechanism, these microorganisms are capable of influencing the expression of genes that favor their colonization or plant susceptibility, leading to a successful infection (Scholze and Boch, 2011; Canonne and Rivas, 2012; Doyle et al., 2013). Because of its high effectivity and specificity, this mechanism was used as inspiration for the development of the TALENs (transcription activator-like effector nuclease) technology, which permits the edition of genomic DNA sequences by targeting nucleases to specific loci (Joung and Sander, 2013). Some other bacterial effectors have been reported to target and modify eukaryotic transcription factors, inducing altered expression of defense genes and ultimately increasing the bacterial fitness (Canonne and Rivas, 2012). Another example of pathogen effectors directly modulating plant gene expression is the PopP2 protein from *Ralstonia solanacearum* that directly acetylates WRKY transcription factors to suppress plant immunity (Le Roux et al., 2015) (**Figure 6**).

There is increasing evidence that plant pathogens, like animal pathogens, can also affect their hosts’ gene expression and defense responses thought the direct modulation of the chromatin fiber. Various studies suggest that bacterial effectors and proteins could directly modify chromatin, but direct evidence for this phenomenon has been obtained only recently. For instance, the expression of the XopD effector of *Xanthomonas campestris* pv. *vesicatoria* was observed to affect the DNA accumulation in nuclear bodies in the plant nucleus, as well as the localization of nuclear proteins in these structures. These results suggested that this effector affects its host chromatin conformation. However, the mechanisms by which this occurs are still not clear, and it has been hypothesized that this SUMO protease may act by targeting one or more chromatin modifiers (Miller et al., 2010; Canonne et al., 2011; Canonne and Rivas, 2012).

The 6b oncoprotein encoded in the T-DNA of *Agrobacterium tumefaciens*, interacts specifically with histone 3 and has the capacity to mediate nucleosome formation *in vitro* (Terakura et al., 2007). Furthermore, transgenic Arabidopsis plants expressing this protein displayed repression of some auxin-inducible genes, including *IAA3/SHY2, IAA6*, and *ACS.* The 6b mutant, lacking its C-terminal moiety, is unable to interact with H3 and to perform its histone chaperone activity. This mutant also fails to promote hormone-independent growth in tobacco cells, indicating the importance of this protein in the pathogenesis of this microorganism (Terakura et al., 2007).

Geminiviruses have also acquired mechanisms that inhibit the silencing of their genome triggered by plant defense. Several mechanisms inhibiting PTGS have been described, but in this review we focus on viral mechanisms of TGS suppression, because they are more directly related to chromatin modifications. The first reported viral protein capable of suppressing TGS was C2 of the Beet Severe Curly Top Virus (BSCTV), which attenuates the degradation of the Arabidopsis SAMDC1 protein and reduces *de novo* DNA methylation of the viral genome (Zhang et al., 2011). Afterward, other viral proteins inhibiting TGS have been described, such as the geminivirus Rep that down-regulates the expression of DNA methyltransferases, decreasing DNA methylation levels in the viral genome and hence TGS (Rodríguez-Negrete et al., 2013). More recently, the geminivirus-encoded TrAP protein was shown to inhibit the activity of the Arabidopsis histone methyltransferase SUVH4/KYP, leading to reduced levels of H3K9me2 and cytosine methylation in both the plant and the viral genome, thereby increasing viral replication (Castillo-González et al., 2015). Altogether, these examples provide evidence for the several and diverse weapons that these viruses have developed in order to cope with the complex layers of plant immunity. Nevertheless, bacterial and viral pathogens are not the only ones that have adapted to manipulate their host epigenetically to favor themselves: some fungal and oomycete effectors have also been found to do this.

Recently, the *Phytophthora sojae* effector PsAvh23 was proven to sequester the soybean ADA2 subunit of the SAGA acetyltransferase complex, inhibiting the interaction of this protein with the catalytic subunit GCN5 (Kong et al., 2017). The expression of *PsAvh23*, or the suppression of *ADA2/GCN5* in soybean plants, led to genome-wide decreased levels of H3K9 acetylation. These changes in histone acetylation correlate with decreased expression of hundreds of genes, including many pathogen-responsive genes such as *WRKY* and *NAC* transcription factors, MAP kinases and heat-shock proteins, and finally to an increased pathogen susceptibility (**Figure 6**) (Kong et al., 2017). The high conservation of the SAGA complex and its subunits among different eukaryotic taxa raises the question of the mechanism that this pathogen uses in order to avoid its own effector protein to target its own HATs and affect transcription on its nucleus. It may be reasonable to hypothesize that the protein remains inactive in the *P. sojae* cells and suffers modifications during its secretion from the fungal cell that activate it, or perhaps is activated by the plant cell machinery itself. However, it would be interesting to explore this in depth in order to increase our knowledge about the evolutionary mechanisms shaping plant–pathogen interactions.

### Epigenomic Regulation of Virulence in Plant Pathogens

In the last years there has been an increasing wave of research focusing on the pathogen side of plant–pathogen interactions, including pathogen epigenetics. Fungal species of the genus *Fusarium* have become models for the study of the epigenomic regulation of virulence. In *Fusarium graminearum*, the mutation of *FTL1*, the homolog of the yeast *SIF2* sub-unit of the Set3 HDAC complex, affected the conidiation, sensitivity to plant defensins and the infection capacity of this cereal pathogen (Ding et al., 2009, 2010). A later study characterized the HDF1 protein, homolog of the Hos2 HDAC and physical interactor of FTL1 in the Set3 complex. Similar to *ftl1*, the *hdf1* mutant presented decreased virulence, together with inability to spread to other infection sites, defects in sexual reproduction and reduced conidiation. Molecularly, the mutation induced a 60% decrease in HDAC activity, together with the misregulation of hundreds of genes, likely responsible for the developmental and virulence abnormalities of the mutant (Li et al., 2011). Histone acetylation has also been found to be crucial for the regulation of genes involved in the production of mycotoxins: for instance, in *Fusarium verticillioides*, the chemical inhibition of HDACs induces the expression of *FUM1* and *FUM21*, two crucial genes in the biosynthesis of class B fumonisin (FB) mycotoxins. In addition, the histone acetylation levels of the promoters of these loci increased in FB-inducing conditions, indicating that histone acetylation is, at least partly, responsible for the control of the FB toxin production (Visentin et al., 2012). In *F. fujikuroi*, a rice pathogen, the HDACs FfHda1 and FfHda2 have been shown to regulate the production of secondary metabolites and virulence (Studt et al., 2013); HDAC activity also seems to regulate infectious growth in fungi from other genera, such as the rice pathogen *Magnaporte oryzae*, where mutants in components of the HDAC complex proteins *TIG1, SET3, SNT1*, and *HOS2* present defects in conidiogenesis, plant invasion, pathogenicity, and an increased sensitivity to ROS (Ding et al., 2010). Likewise, HDAC activity regulates the virulence in *Cochliobolus carbonum* on maize and the infection capacity of *Rhynchosporium commune* on barley, suggesting an importance of histone acetylation dynamics in the adaptation of fungi from diverse phyla to a pathogenic lifestyle (Baidyaroy et al., 2001; Siersleben et al., 2014).

Like histone acetylation, histone methylation has also been described as an important mechanism for the regulation of virulence in *Fusarium* species and other fungi. For instance, the KMT6 methyltransferase of *Fusarium graminearum* was found to regulate the expression of gene clusters that participate in the biosynthesis of secondary metabolites (SMs). The *kmt6* mutant, apart from presenting several developmental problems, displays complete lack of the H3K27me3 repressive mark and constitutively active expression of mycotoxins and pigments (Connolly et al., 2013). The Ccl1 subunit of other *Fusarium* complex with methyltransferase activity, COMPASS, was shown to regulate genome-wide H4K4me3 levels. Even though its mutation did not lead to any developmental abnormalities, the SMs production under inductive conditions was compromised. However, and unexpectedly, such production was restored to wild-type levels upon infection, and the mutation did not have an impact over virulence (Studt et al., 2017).

## Concluding Remarks and Perspectives

The present review aimed to illustrate the importance of chromatin remodeling in the regulation of plant responses to pathogen infection, as well as its role in the regulation of virulence in pathogens from diverse phyla. We highlighted striking examples of how histone modifications, histone variants and chromatin remodelers impact the expression of defense genes, permitting plants to mount a pertinent immune response. Over the years, research groups have identified key genes, involved at various levels -from pathogen perception to defense responses itself-, in disease susceptibility or resistance. However, it has become clear that chromatin remodelers and modifying enzymes represent crucial nodes in the complex regulatory networks behind immunity and development. Interestingly, research in human health has also provided evidence for the important role played by histone modifications in immunity, even when vertebrates rely on an acquired immune system that is responsible, to a great extent, of immune memory. There is growing proof that the mammalian innate immune system, like the plant one, depends highly on epigenomic processes and elements, such as histone modifications and non-coding RNAs (Mehta and Jeffrey, 2016; Araki and Mimura, 2017). Such observations may suggest that the same phenomenon may occur along diverse taxa within the eukaryotes. For this reason, we consider highly interesting to examine such processes under an evolutionary perspective, in order to determine whether there is an existing relation between the innate immune system and epigenomic network complexities among different groups of organisms.

It has become clear that it is crucial to understand how genomes are regulated in response to stress. To this end we need more genome-wide data to understand what the global changes induced by mutations in genes involved in chromatin remodeling are. It is not just about controlling the expression of a few genes, but about conditioning entire genomes for a fast and strong response to specific stresses. Currently available studies have proven the vertical transmission of genomic traits through a limited number of generations (Luna et al., 2012; Rasmann et al., 2012; Slaughter et al., 2012; Ramírez-Carrasco et al., 2017). In this context, we consider fundamental to generate new research in order to highlight the extent to which stress-induced epigenomic reprogramming can be transgenerationally inherited in plants. Together with high-throughput technologies, these data could provide us with bigger insights into the epigenetic (*sensu stricto*) aspects of plant immunity. We envision that finding out which chromatin remodeler does what, when and how -in other words deciphering the chromatin dynamics during stress-, can allow us to fine-tune the plant responses to environmental cues. One could then engineer tailored epigenomes and ultimately win the battle against pathogens.

## Author Contributions

JR-P and SP wrote the manuscript. CR performed the writing of some sections, participated in the proofreading and contributed with intellectual input. AB, HH, and MB contributed intellectually and MB coordinated the manuscript preparation. All authors read and approved the final manuscript.

## Conflict of Interest Statement

The authors declare that the research was conducted in the absence of any commercial or financial relationships that could be construed as a potential conflict of interest.
